# The emotional and mental health needs of young carers: what psychiatry can do

**DOI:** 10.1192/bjb.2019.78

**Published:** 2020-06

**Authors:** Roswitha Dharampal, Cornelius Ani

**Affiliations:** 1 Tavistock and Portman NHS Foundation Trust, UK; 2 Surrey and Borders Partnership NHS Foundation Trust and Imperial College London, UK

**Keywords:** Young carers, mental health, psychiatry, systematic review

## Abstract

**Aims and method:**

To review the literature on the emotional and mental health needs of young carers of parents with mental illness and the extent to which such needs are recognised and supported by professionals. Three databases were systematically searched from 2008 to 2018, and five studies met the inclusion criteria.

**Results:**

The key findings were that young caregivers had a significantly higher dose-response mortality risk than their peers; were at increased risk of mental health difficulties, especially where the ill family member was a parent and had mental illness or misused substances; were overlooked by professionals owing to a lack of awareness; but could derive benefits from their caring role when appropriately supported.

**Clinical implications:**

Young carers are at increased risk regarding emotional and mental health needs; this risk could be mitigated by professionals recognising the young carer's role and including them in their parent's treatment plan.

## Background

Societal awareness of young carers and the potential effects of caring on their health and development has increased in the past 20 years.^1^ A ‘young carer’ is ‘a child or young person under 18 who provides regular or ongoing care and emotional support to a family member who is physically or mentally ill, disabled or misuses substances’.^2,3^ The 2011 Census in England and Wales showed that 166 363 children in England cared for their parents, siblings or family members, an increase of 20% on the number recorded in the 2001 Census.^4,5^ However, this was thought to be an underestimate.^2^ The prevalence of informal caring in the underage population was estimated as a minimum of 2–4% in Western countries.^6^

Dearden and Becker reported that most young carers cared for parents, particularly mothers, although some provided support for grandparents, siblings or other relatives. Tasks undertaken by young carers included housework, general healthcare – for example, assisting with mobility or giving medication – intimate personal care such as bathing or toileting, and emotional support.^7^ Emotional support by young carers was more likely to be offered to parents with a mental health problem.^7–10^ Caring responsibilities ranged from a few hours a week to over 100 in extreme cases.^11^

Half of all conditions supported by young carers involved physical health, but almost a third (29%) were for mental health problems, while 17% related to learning difficulties and 3% were for sensory impairments.^7,12^ Extrapolating from these figures suggested that 55 000–60 000 children in the UK cared for a parent with mental illness.^13^ According to 2018 estimates for England, 3.7 million children aged 0–17 (31.7%) were in a household where a randomly selected adult had at least moderate mental health symptoms, including 1.6 million (13.5%) where the adult had severe mental health symptoms.^14^

For psychiatrists, the young carers most likely encountered in work are those of their adult patients with mental illness. Estimates in Australia and the USA of the proportion of people accessing mental health services who were parents ranged from 20 to 60%.^15–18^ In the UK, the majority had common mental disorders such as depression or anxiety, but some (0.5%) had a psychotic condition.^19^ According to recent UK estimates, parents comprise at least 25% of adult mental health patients with significant interpersonal/personality difficulties, including 63% of women with psychosis and 25% of adults in acute psychiatric hospital settings.^20^

The 2011 Census in England and Wales showed that one in eight young carers was under the age of ten, and some were as young as five.^5^ According to Canadian research among youth aged 15–24 years, females accounted for the majority of carers, and there was an increasing feminisation of care as youth aged, the differences being most acute at the highest care levels.^21^ It was suggested that this pattern was similar to that in the UK and Australia.^7,10,21^ A further UK survey suggested that young carers were 1.5 times more likely than their peers to be from Black, Asian or minority ethnic communities, and twice as likely to not speak English as their first language. They were 1.5 times more likely than their peers to have a special educational need or a disability.^11^

How the young carer and parent were viewed often depended on the disability or diagnosis, such that having parents with mental health problems was presumed to pose more adverse challenges.^1^ Young carers were often considered as a homogenous group; however, it has been argued that those caring for a mentally ill parent could experience particular risks and needs. Studies from several countries have found that children of parents with a mental illness were at a greater risk of poorer outcomes than their peers,^22,23^ with higher rates of mental illness^24^ and poorer development in behavioural,^25,26^ social,^27^ and academic^28^ domains.^22^ According to Pakenham and Cox, the presence of any family member with an illness was associated with a greater risk of mental health difficulties for young people, relative to their peers from ‘healthy’ families. This risk was further elevated if the ill family member was a parent and had mental illness or abused substances.^29^ In the UK, parental mental ill health was a significant factor for children entering the care system.^30^

Aggregated data suggested that a child had a 30–50% chance of developing a serious mental illness if they had two parents with mental illness.^31^ A child of a parent with an affective illness had a 40% chance of developing an affective disorder by age 20, compared with a 20–25% risk in the general population.^32–35^ However, the increased risk noted in these studies may have a multifactorial aetiology.

It has also been argued that younger carers can sometimes overcome the effects of extreme adversity^36^ with information, recognition of their role, and inclusion in their parent's treatment plan. Studies suggest that some young carers may even derive some benefit from their caring role.

## Aim

The main aim of this literature review was to explore the emotional and mental health needs of young carers and their circumstances, particular those related to their parents' mental health. The review also explored the extent to which the needs of young carers were recognised and supported by the psychiatrists and other professionals working with their parents.

## Method

The PRISMA guideline^37^ was followed to search three databases (EMBASE, Medline and PsychInfo), which are accessible from the Royal College of Psychiatrists library. The search covered 2008–2018 using the terms ‘emotional health or emotional stability or psychological health or social psychology or mental health’ and ‘young carers’ or ‘young and carers’ or young caregivers or ‘young and caregivers’. Boolean operations and truncations were employed to allow for alternative endings in the keyword searches. The search was limited to the past 10 years in order to focus on more recent developments in the field, which are more likely to be relevant to current practice. Additional literature was accessed through contact with the authors of some of the papers, a charity that supports young carers, and references from retrieved papers.

**Fig. 1:**
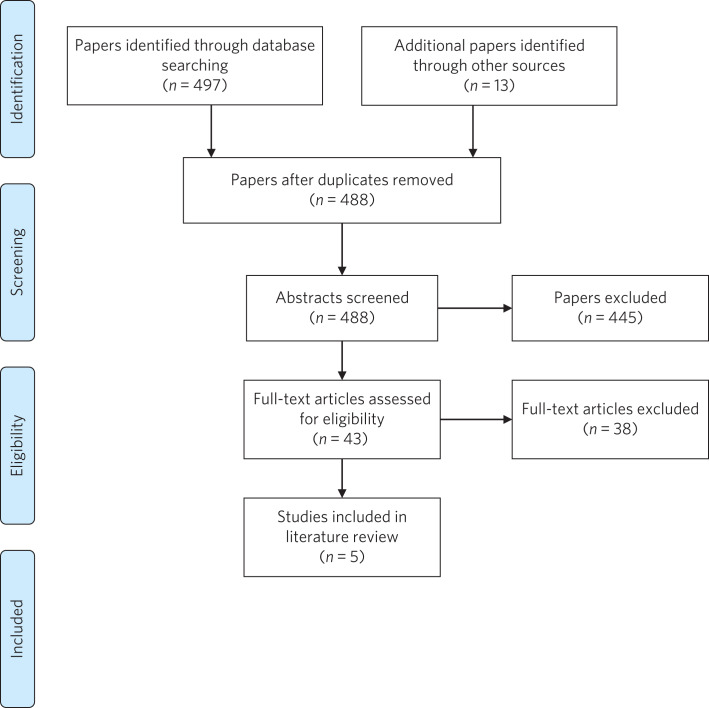
Study selection

Studies were included if they were published in English and involved a primary study published in the past 10 years that identified the emotional and mental health needs of young carers and had a minimum of 14 participants. The latter criterion was based on the fact that very small sample sizes would limit the generalisability of results to the wider young carer population.^38^ Four hundred and eighty-eight abstracts were identified; initial screening identified 43 relevant studies, whose full texts were assessed. Finally, five studies were selected (Fig. 1).

## Results

The literature review focused on five recent papers published between 2013 and 2018 which investigated the emotional and mental health needs of young carers, the circumstances which gave rise to them, current psychiatric practice and possible improvements. One study^39^ was census-based, retrospective and longitudinal. The others, reflective of the majority of studies in the search, were qualitative and recorded the experiences of young carers. The papers showed the increased and particular needs and risks of young carers of a parent with mental illness, with one study identifying what it referred to as a ‘young carer penalty’.^38^ The census-based study further found a higher mortality risk among young carers.^39^ The shortcomings of mental health services in their consideration of young carers were also raised. The studies are summarised in Table 1.

**Table 1:** Summary of studies reviewed

Citation	Study design, country and sample	Results	Conclusion
Leu *et al*^54^ (*n* = 30)	Semi-structured interviews Switzerland 16 young carers aged 10–17 years 14 young carers aged 18–25 years	Tasks depended on nature of illness/impairment and availability of other family carers. Intensity varied from part- to full-time responsibility	Highlighted importance of communication with family, professionals and peers
Millenaar *et al*^53^ (*n* = 14)	Semi-structured interviews The Netherlands 14 children aged 15–27 years living with a parent with young-onset dementia (YOD)	Divided into three themes that demonstrated effects of dementia on daily life, different ways of coping, and children's need for care and support	In addition to practical information, more accessible and specific information about diagnosis and course of YOD needed to provide better understanding for children. Underlined need for personal, family-centred approach.
Packenham and Cox^29^ (*n* = 2474)	Questionnaire Survey Australia 2474 youth aged 9–20 years (‘healthy family’ *n* = 1768, parental illness *n* = 336, other family member illness *n* = 254, both parental and other family member illness *n* = 116)	Presence of any family member with illness associated with greater risk of mental health difficulties for youth. Risk elevated if ill family member is parent and has mental illness or substance misuse	Serious health problems within household adversely affect youth adjustment
Stamatopoulos^38^ (*n* = 15)	Two focus groups and one in-depth interview Canada 15 young carers aged 15–19 years	Evidence for unique ‘young carer penalty’	Ongoing youth caregiving constitutes a form of hidden labour that carries with it a range of benefits and penalties
Tseliou *et al*^39^ (*n* = 19 621)	Census-based mortality linkage study Northern Ireland Caregivers aged 5–24 years	Young caregivers more likely than non-caregiving peers to report chronic poor mental health. They also differed from older caregivers and were at significantly higher mortality risk than peers. Dose-response relationship between hours devoted to caregiving and mortality risk evident	Young caregivers at significantly increased risk of poor health outcomes

A study by Pakenham and Cox examined differences in adjustment between children of a parent with an illness and peers from ‘healthy’ families, controlling for whether a parent or non-parent family member was ill, the illness type, demographics and caregiving.^29^ The study was questionnaire-based and had a total sample of 2474 youths, comprising those from ‘healthy’ families = 1768 and those from families with parental illness = 336, other family member illness = 254, and both parental and other family illness = 116. The youths, aged 9–20 years, were recruited in Queensland, Australia, mostly from schools. However, recruitment also took place through church groups (*n* = 35), scouts (*n* = 23), university vacation care (*n* = 13), and a young carer association (*n* = 42) in order to achieve more diversity in the sample.

The youths completed questionnaires to obtain information on their age, gender, home postcode, employment, dual- versus single-parent family, number of siblings and ethnic background. An eight-item family responsibilities subscale of the Young Carer of Parents Inventory^40^ was used to assess youth caregiving. A range of positive and negative youth adjustment outcomes were assessed by behavioural-emotional-social difficulties, somatisation and health. Positive adjustment outcomes were assessed by family satisfaction, life satisfaction, positive affect and pro-social behaviour.

The results showed that the presence of any family member with an illness was associated with greater risk of mental health difficulties for youths compared with their peers from ‘healthy’ families. Using Cohen's effect size conventions, the effect sizes for the significant outcomes for parental illness group ranged from small (0.22) to very large (1.0), but they were uniformly small for the ‘other family members’ illness group (range 0.08–0.18). This risk of poorer adjustment was elevated if the ill family member was a parent and had mental illness or misused substances. The latter risk held even when caregiving and demographic factors were considered. Incidentally, caregiving itself was associated with poorer adjustment in six of the seven outcomes considered, even after controlling for illness type and a range of sociodemographic factors.

Parental illness and illness in other family members were both significantly associated with more negative outcomes compared with ‘healthy’ families for all but two outcomes. The effect of ‘parental illness’ was significantly larger than for ‘other family member’ illness for all but one outcome, although the effect sizes were small. However, as these data were cross-sectional, the associations may not be causal, as it is also possible that the additional risk attributed to caregiving may be confounded by other unmeasured factors such as genetic influences.

A recent study^39^ investigated the association between caregiving and health/mortality risk in young caregivers when compared with non-caregiving peers and older caregivers. They used a census-based record to link all residents enumerated in the 2011 Northern Ireland Census with subsequently registered deaths data, until the end of 2015. Among those aged 5 to 24 years in the 2011 Census, approximately 4.5% were reported to be caregivers. The presence of a chronic physical and/or mental health condition was measured through the Census, and all-cause mortality was assessed by official mortality records.

This study found that young caregivers had a significantly higher mortality risk than their peers (adjusted hazard ratio = 1.54, 95% CI: 1.10, 2.14). A dose-response relationship between the hours devoted to caregiving duties and mortality risk was evident. Young caregivers were also more likely to report chronic mental health problems than their non-caregiving peers (adjusted odds ratio (OR) = 1.44, 95% CI: 1.31, 1.58).

Further, young carers differed from older caregivers, with the odds of reporting poor mental health inversely related to age. Tseliou *et al* maintained that although the majority of studies found that caregiving may be associated with poor mental and emotional health,^41–44^ this had been moderated by growing recognition that older caregivers had a reduced mortality risk compared with non-caregiving peers.^45–52^ At older ages, less intense caregiving was associated with a reduced risk of chronic poor mental health. However, by 25–44 years old, this was reversed such that a positive dose-response association was observed between caregiving status and mental ill-health. This adverse effect was most evident among 5–17-year-olds. In this younger cohort, those providing more intensive caregiving were more than twice as likely as non-caregiving peers to have poor mental health (adjusted OR = 2.46, 95% CI: 1.70, 3.56).

Light caregiving may be associated with a positive effect on physical health, such as fewer chronic mobility problems compared with non-caregivers.^39^ However, this apparent benefit of caregiving was not observed among younger carers providing higher levels of care. The authors hypothesised that the protective effect of light caregiving, especially for older carers, could be due either to the physical requirements of the caregiving role or an instance of ‘selection into the role’ by healthier individuals.^39^ To further illustrate the differential effect of care givers' age, the authors stratified the data by age group (young adults versus children and adolescents) and found that although less-intensive caregiving in the older cohort was associated with 35–40% reduced odds of reporting chronic mobility problems, those aged 5–17 were more likely to report mobility problems (OR = 1.61, 95% CI: 1.16, 2.23).^39^

Although the census-based study by Tseliou *et al* had unsurpassed population coverage and encompassed many hard-to-reach groups, it was nonetheless limited by the fact that it may have missed a disproportionate number of young adults and caregivers in deprived inner-city areas. Also, the proxy nature of census returns makes it likely that the parent or guardian completed the ‘self-assessed’ health question, which might have led to confounding, although not for mortality risk.

The literature search identified a study which explored the experiences and needs of children living with a parent with young-onset dementia.^53^ The study recruited 215 patients and their families through memory clinics, regional hospitals, mental health services and specialised day care in The Netherlands. For ethical reasons, inclusion was restricted to children older than 14 years. There were a total of 35 eligible participants living in 29 families, of which 15 agreed to participate. The method involved semi-structured interviews with 14 of the young people, six males and eight females, aged between 15 and 27, with an average age of 21 years. In three families, the mother had dementia. The mean age of the parent was 53.6 years. Five of the parents had Alzheimer's disease, four had frontotemporal dementia, one had vascular dementia and one had dementia not otherwise specified. Most of the parents had mild to moderate dementia. The type of care the young people provided included housekeeping tasks (cooking, cleaning, and grocery shopping), supervision and social contact.

Semi-structured interviews were analysed using a qualitative inductive content analysis, which revealed three major themes. The first theme indicated the effects of dementia on daily life, including changing relationships within the family, children's difficulties managing responsibilities while maintaining a life of their own, and children's concerns about their future. The second theme reflected the different ways the children coped, including acceptance, avoidance, searching for relief and actively dealing with changes. The third theme revealed the need for care and support. Many children wanted to know more about dementia but received little information after the diagnosis from either their parents or healthcare professionals. In addition, they wanted practical guidance in dealing with their parent.

However, the study by Millenaar *et al* was limited by the less-than-optimal representativeness of the sample due to the restricted availability of children in the target population and high refusal rates. The authors acknowledged that these sampling difficulties may have led to an underestimation of needs, because those who were not included may have found it too demanding to participate in the study or too difficult to talk about their needs.

A qualitative study with young carers and young adult carers in Switzerland further explored the role of communication with professionals about the caring situation.^54^ Interviews were conducted with 16 carers aged 10–17 years and 14 aged 18–25 years. The interviews were recorded, transcribed and analysed following a grounded theory approach.

The study reported that the participants found talking to professionals was often seen as difficult. The young people described situations in which they were simply overlooked by experts from healthcare or social services. It was recognised this generally did not happen because of malicious intent by professionals, but rather owing to a lack of awareness about young carers and their roles and responsibilities.^55^ In particular, getting into contact with healthcare professionals in hospitals seemed to be challenging when young people were the main carer. Information was often withheld by healthcare personnel owing to a perceived need for confidentiality. Some young adult carers had to resort to alternative ways, for example, the internet, to access necessary information when neither professionals nor the care-receiving family member provided it.

In the final study selected for this literature review, a qualitative focus group design was chosen to explore the ‘lived realities’ of young people providing unpaid familial caregiving in Canada.^38^ Two focus groups and one in-depth interview were held with 15 young people aged 15–19 years, who were current or past members of a formal young carers programme. Participants also completed a short survey after the discussion, representing a form of ‘concurrent triangulation’. Purposive sampling was used to recruit young carers.

Over half the youth provided care primarily to a sibling, with the next largest group caring for a parent, and several caring for multiple family members. The main conditions ranged from substance (alcohol) abuse to terminal cancer to autism, and a high likelihood of comorbidity existed. A diverse range of ethno-racial profiles were captured, with just under half the participants self-identifying as Caucasian and the remaining as Arab, Black, South Asian and Chinese. However, gender imbalance was evident, with only three male young carers in the sample. The author maintained that this partly reflected the conflict boys experienced when discussing or recognising their care contributions. ‘This gendered reticence by young men due to the presumed violation of expected gender roles makes it more difficult for them to get recognition and receive dedicated support as carers’.^38^

The young carers derived a range of positive benefits, including added maturity, empathy and the strengthening of familial bonds. However, they also incurred a range of difficulties that caused short- and long-term harm to their personal and professional development Together, these difficulties presented what the author referred to as a ‘powerful young carer penalty’, a term used to highlight the personal (emotional, familial and social) and professional (education and employment-based) disadvantages incurred by the young person's substantial and ongoing caregiving.^38^

## Discussion

The main aim of this literature review was to investigate the emotional and mental health needs of young carers and their circumstances, particular those relating to their parents' mental health. It also explored the extent to which the needs of young carers are recognised and supported by psychiatrists and other professionals working with their parents, with shortcomings raised. The review concentrated on five papers that showed the increased and particular needs and risks of young carers of a parent with mental illness. One even found a higher mortality risk among young carers. The findings are further explored here.

According to Pakenham and Cox the type of illness present in the home was associated with differential adjustment outcomes, with mental illness and substance problems associated with more negative adjustments across a range of outcomes.^29^ They suggested a potential explanation for these findings: that in general, compared with physical illness, mental illness and substance misuse were less understood in the community and more likely to be associated with greater social disadvantage, unpredictability of symptoms, family and social disruption, stigma, discrimination and parent-child attachment difficulties.

These results are similar to findings from previous studies. Cooklin and Hindley suggested that parental mental illness could be responsible for serious interruptions in a child's cognitive and emotional development.^56^ They cited a list of adversities faced by children affected by parental mental illness, which could affect their emotional life, attachment and development. These included the ill parent's disordered thinking and behaviour, the loss of emotional closeness and the witnessing of distressing side-effects of treatments.^56^ According to Mechling, many children witnessed or had to assist their parent in a mental health crisis, such as a suicide attempt, psychotic episode or aggressive state,^57^ leading other authors to highlight that this was a responsibility beyond young people's years.^8,58,59^

Millennaar and colleagues discussed the influence on the daily lives of children of parents with young-onset dementia. Children felt that the child-parent bond was inverted as their parent became increasingly dependent.^53^ There was more tension at home due to the stress of the caring process and changes in the parent with dementia. They witnessed strain on a healthy parent, had difficulties adjusting to the behavioural, cognitive and personality changes in the parent with dementia, and had to contribute more to the household. Millennaar *et al* also suggested that parents of young carers were not always aware of their child's needs.

Similarly, according to Stamatopoulos, substantial caregiving by young people affected their joint familial, social and emotional well-being. Numerous young carers revealed strained familial bonds, often linked to a real or perceived inequality in the provision of care, in addition to limited opportunities for socialisation outside the household. A heightened sense of missing out created strain on existing friendships and was tied to an inability to partake in social outings outside school.^38^ Unbalanced friendships, resulting from parental-role spillover, further strained young carers' existing peer network.

Pakenham and Cox hypothesised that the elevated mental health problems in children of a parent with illness relative to those from healthy families were due to their extra caregiving demands.^29^ However, they found that the effects on youth adjustment of a family member with serious illness were not attributable in the main to factors such as differential access to community services, being in a sole parent family, the age or gender of the youth, or increased caregiving responsibilities, although all these factors were implicated in adjustment outcomes. They acknowledged that the absence of significant interactions in their results was not consistent with a previous study,^60^ where a complex set of potential moderators including age of children, socioeconomic status and single parenthood were identified. Stamatopoulos also found that the severity of the ‘young carer penalty’ was associated with two key factors: family size and type of condition requiring care. Specifically, participants from single-parent and single-child families generally incurred a greater ‘penalty’, as did those providing care in the context of more stigmatised illnesses such as mental illness and substance abuse, and/or more debilitating physical illness or disability.^38^

In their study on the variation of caregiver health and mortality risk by age, Tseliou *et al* proposed a number of reasons that caregiving may be deleterious to the health and well-being of young carers.^39^ Providing care could have interfered with schooling and the formation of healthy social networks, thereby creating issues with other aspects of social and emotional development and leading to a problematic transition to adulthood. The authors suggested that many of the positives associated with caregiving at older ages may not hold true for younger ages. At older ages, where activities such as paid employment no longer applied, caregiving may provide a purposeful role that tightens interpersonal bonds appropriate to expectations of both age and existing relationships, and may be seen as a natural progression, with positive caregiving attributes being associated with lower mortality risk. At younger ages, the expectations of role relationships and function are different, and significant caregiving responsibilities are likely to be at variance with perceived social norms. In contrast to older ages, young caregivers may feel constrained in undertaking a role they had little choice in accepting and that they considered inappropriate for their age. The authors highlighted that the feeling of duty to provide care has been linked to high caregiver burden and worse outcomes among child caregivers.

Leu *et al* raised the failure among professionals to identify young carers and some potential causes of their health and emotional difficulties.^54^ Although many parents may have had an earlier diagnosis of, for example, depression, bipolar disorder, schizophrenia or personality disorder, they may later have gone unnoticed by services.^57^ A secure attachment between child and parent could have been undermined if the parent was inadequately treated or supported.^61^ When treatment did occur, clinicians may not have asked whether the adult client had children, and, if parental status was discussed, the focus of treatment was on the adult and the child's needs may have been overlooked.^57^ Millenaar *et al* found that professionals in contact with families often failed to identify children as providers of informal care because they did not ask about their caring role.^53^ A Royal College of Psychiatry report recognised that professionals may have agreed that offering support to the children of their patients was important but often felt this was not their role, and their natural sympathy and alliance with their patient may have led to ‘blindness’ about the needs of the child.^33^ Staff in mental health teams or in-patient services may have seen themselves as solely the ‘patient's person’.^62^ Some may have seen it as above or outside their expertise or responsibility, and therefore the province of someone else's responsibility.^62^ Wolpert *et al* maintained that the needs of young carers had been traditionally overlooked, falling between adult and child mental health services. However, Child and Adolescent Mental Health Services only see a small proportion of children affected by parental mental illness.^62^

Cooklin suggested that for some young carers, the involvement of services can sometimes worsen aspects of their experiences. Even though a young carer had often been managing the situation for months, no one asked their advice, what they knew about their parent's illness, or what made it better or worse.^13^ Ali *et al* found that young carers received several kinds of information about mental illness and advice about what to do as long as the person with the mental illness was within the healthcare system, but as soon as they were discharged, support for the young carer ended too.^63^ Despite the statutory requirement in the UK that mental health services elicited the views of children and young people about the care plans for their parents' treatment, relatively few were talked with directly about the nature of the illness.^62^ McAndrew *et al* cited young carers' experience of their relative being discharged from hospital but no one explaining about changes in their medication, the administration of which had previously been the young carer's responsibility.^30^ Similar to other young carers, some children of parents with young-onset dementia were not included in conversations with healthcare professionals after diagnosis.^53^

Not all children of parents with a mental illness reported poor outcomes.^22,38^ Dearden and Aldridge maintained that there were positive aspects of caring for children and parents, as long as support services were in place which adequately addressed the needs of all family members. These positive aspects included enhanced maturity, responsibility and independence, life skills, increased understanding about disability issues and stronger family ties.^64^ According to Fraser and Pakenham, this led to the development of a resilience model whereby the potential harmful effects of risk factors were mitigated by the influence of protective factors. Interventions should therefore focus on targeted modification of risk factors such as isolation, while promoting protective factors including independence and psychosocial skills.^22^

Bilsborough reported ten demands of mental health professionals by young carers, of which the top three were (a) introduce yourself, (b) tell us who you are and what your job is, and (c) give us as much information as you can.^65^ Cooklin identified what children said they needed: a two-way explanation of the parent's illness’ access to a neutral adult with whom the child could discuss the illness and contact in times of crisis, and who could act as the child's advocate; an opportunity for the child to address their fears that they would ‘catch’ or that they ‘caused’ the illness or that the parent may die from it and/or they might not see them again; interventions to diminish the child's social isolation, to learn they are not the only one with the problem, and to allow them to meet other young people with similar experiences; and rebalancing the child's inverted role as carer, including opportunities to do childish or youthful activities with other young people and sharing the load of responsibility with one or more adults.^62^ However, Cooklin warned of the dangers of offering counselling unless the child explicitly accepted the idea of therapeutic help, as this might increase their unwelcome sense of identification with the ill parent and define them as on a similar pathway.^62^

It was noted that family work or therapy has become more available within adult mental health services.^62^ Thus, a ‘whole family approach’ model, developed in the UK has begun to be the dominant paradigm internationally. In an NHS England presentation, Cooklin recommended that all psychiatric adult care plan assessments should establish: knowledge of all children face to face; who if anyone has assessed the needs of the child or the effects of the parent's illness on them, and what if any referral needs to be made; and who has explained the patient's illness to the child.^56^

In the UK, the Care Act 2014 and Children and Families Act 2014 provided statutory recognition for young carers' entitlement to regular assessments of their ability and suitability to provide care, and, importantly, of the effects of the role on them.^66^ Of note, the UK alone was classified as ‘advanced’ in an international comparison of awareness and policy responses to young carers.^67^ The UK was advanced in terms of awareness, research, law, social policy, government guidance and service delivery. No country was identified as having developed extensive awareness or sustained or sustainable policies. The analysis noted that the reality in most countries is that young carers still fall through gaps in policy and legal safety nets.

## Limitations

The main limitation of studies in this review was the absence of intervention studies relating to young carers. Most of the studies were qualitative and recorded the experiences of young carers and professionals in contact with them. Although the qualitative studies were limited by small sample sizes, resulting in poorer generalisability of their findings, they had the strength of seeking out more detailed and in-depth accounts.^21^ The review was limited by searching only three main databases. Also, the search was limited to recent papers published in the past 10 years. Finally, only the first author applied the selection criteria.

## Conclusion

This literature review found that children and adolescents who cared for a parent with illness may be at higher risk of a range of emotional and mental health needs. Those who specifically care for a parent with mental illness could be at an increased risk, possibly owing to the potential for a more disordered relationship with the parent as a result of their mental illness. However, it is argued that such adversities could be mitigated with the help of information, recognition of the young carer's role, and their inclusion in the parent's treatment plan. Internationally, the UK appears to afford the most recognition to young carers. However, many young carers in the country are still falling through the net because mental health professionals who treat their parents fail to recognise the significance of young carers. Changes in practice to address this are crucial.

## About the authors

**Roswitha Dharampal** was the Dinwoodie Research Fellow for Children First, Royal College of Psychiatrists, and is ST6 in Child and Adolescent Psychiatry, Tavistock and Portman NHS Foundation Trust, London, UK; **Cornelius Ani** is Honorary Clinical Senior Lecturer, Division of Psychiatry, Imperial College London, and Consultant in Child and Adolescent Psychiatry, Surrey and Borders Partnership NHS Foundation Trust.
